# *In silico* and preclinical drug screening identifies dasatinib as a targeted therapy for T-ALL

**DOI:** 10.1038/bcj.2017.87

**Published:** 2017-09-08

**Authors:** S Laukkanen, T Grönroos, P Pölönen, H Kuusanmäki, J Mehtonen, J Cloos, G Ossenkoppele, B Gjertsen, B Øystein, C Heckman, M Heinäniemi, M Kontro, O Lohi

**Affiliations:** 1 Tampere Center for Child Health Research, University of Tampere, and Tampere University Hospital, Tampere, Finland; 2 Institute of Biomedicine, School of Medicine, University of Eastern Finland, Kuopio, Finland; 3 Institute for Molecular Medicine Finland FIMM, University of Helsinki, Helsinki, Finland; 4 Department of Pediatric Oncology/Hematology, VU University Medical Center, Amsterdam, The Netherlands; 5 Department of Hematology, VU University Medical Center, Amsterdam, The Netherlands; 6 Department of Clinical Science, Hematology Section, University of Bergen, Bergen, Norway; 7 Department of Internal Medicine, Hematology Section, Haukeland University Hospital, Bergen, Norway; 8 Department of Hematology, Hematology Research Unit Helsinki, University of Helsinki, and Helsinki University Central Hospital Cancer Center, Helsinki, Finland

Systematic development of combination chemotherapies has significantly improved the prognosis of acute lymphoblastic leukemia (ALL). Patients with T-cell ALL (T-ALL) still have less favorable outcomes, and the prognosis of relapsed T-ALL is dismal.^1^ In order to find novel targeted therapies for T-ALL, we retrieved the gene expression profiles of 4430 leukemia samples from the Gene Expression Omnibus (GEO) and performed an *in silico* drug target screening, where the expression profiles were compared with known drug targets in the Drug signature database (DsigDB).^2, 3^ This compound library contains both FDA (Food and Drug Administration)–approved and novel investigational drugs. Strong expression of Src family tyrosine kinase *LCK* was detected in T-ALL samples, exceeding that of other leukemias and normal T cells (Figure 1a), whereas a LCK-targeting drug, dasatinib, decreased the kinase activity of LCK to 1% in comparison with the control at a 100 nM concentration.^4^ Dasatinib is known to have multiple intracellular targets, and we noticed that some of them had a similarly elevated expression in T-ALL patients (Figure 1b and Supplementary Figure S1). Therefore, our combinatorial drug/target screening suggests dasatinib as a candidate targeted therapy for T-ALL patients.

To experimentally evaluate the potency of dasatinib, we treated T-ALL cell lines with increasing concentrations of dasatinib (1–1000 nM). After 72 h, the most significant response was seen in Jurkat cells, with a 31% viability decrease at a 10 nM concentration (*n*=3, *P*=0.0039; Figure 1c and Supplementary Figure S2). As dasatinib inhibits several kinases that are key regulators of cellular proliferation and viability, we chose a panel of likely candidates (9 targets based on the *in silico* screening and 12 other well-known targets from the literature), and analyzed their expression in T-ALL cell lines by quantitative reverse transcriptase-PCR (RT-qPCR) and western blotting. *LCK* was the most prominently expressed gene in T-ALL cell lines, whereas *FYN*, *ABL1*, *MAP2K5*, *MAP4K5* and *LYN* were expressed at lower levels (Figure 1d and Supplementary Figure S3). Knockdown of *LCK* in a dasatinib-sensitive cell line (Jurkat) significantly decreased cell proliferation (14% decrease, *P*=0.0289, *n*=7, Figure 1e), whereas knockdown of *FYN*, *ABL1*, *MAP2K5* and *MAP4K5* had no significant effect (Supplementary Figures S4a–d). Importantly, Jurkat cells with reduced LCK activity due to a deletion of exon 7 (cell line J.CaM1.6) lost dasatinib sensitivity (Figure 1f). Moreover, *LCK* knockdown did not cause statistically significant decrease of proliferation in relatively dasatinib-insensitive P12-Ichikawa cell line (Supplementary Figure S4e). These results suggest that LCK is the prime target of dasatinib in T-ALL.

We next performed *ex vivo* drug testing of 22 primary T-ALL samples. In 6 cases (27%), the response to dasatinib was significant based on drug sensitivity scores (DSS, using a cutoff value of 10, Figure 2a).^5^ Half-maximal growth inhibition concentrations (IC_50_) ranged between 1.3 and 16 nM, whereas the control samples had an IC_50_ of >1000 nM (Supplementary Figure S5). We also noted a negative correlation between dasatinib and glucocorticoid DSS scores (Supplementary Figure S6). Previously, dasatinib sensitivity has been reported in T-ALL cases with *NUP214-ABL1* fusion.^6, 7, 8^ In contrast, none of the dasatinib responders in our sample set carried the fusion gene based on either genomic PCR or RNA-sequencing analysis (Supplementary Figure S7). *LCK* was strongly expressed in four out of the five dasatinib-responsive patient samples, whereas the expression of other potential targets varied from a low (*LYN*, *ABL1)* to medium level (*FYN*, *MAP2K5* and *MAP4K5*, Supplementary Figure S8a). As *LCK* was also relatively strongly expressed in dasatinib-insensitive patient samples, no correlation between dasatinib response and *LCK* expression was observed (Supplementary Figure S8b).

As T-ALL subgroups can be separated by expression of specific transcription factors, we explored whether dasatinib sensitivity was associated with any specific subgroups.^9^ In addition to Jurkat cells that belong to the TAL1 subgroup, five out of six dasatinib-sensitive patient samples showed either prominent *TAL1* expression (Figure 2b) or carried *SIL-TAL1* fusion (data not shown). There was also increased expression of *LMO2* and *HOXA9/10* genes in some samples (Supplementary Figure S9). 6q deletions have also been associated with the TAL1 subgroup, and three out of six samples carried the 6q deletion in cytogenetic analyses (Supplementary Table S1).^9^ When *LCK* expression was correlated with the subgroup information in our GEO-based gene expression data set, a statistically significant correlation was found between *LCK* expression and the TAL1 subgroup (Figures 2c and d and Supplementary Figure S10). However, no direct transcriptional regulation of *LCK* by TAL1 was seen when the expression of *TAL1* was knocked down (Supplementary Figure S11, data reproduced from Sanda *et al.*^10^).

Taken together, the combination of *in silico*, *in vitro* and *ex vivo* data indicate that dasatinib exerts clinical utility beyond the originally suggested *NUP214-ABL1* cases that only represent 4–10% of T-ALL patients.^6, 11^ Our results are in line with the data by Frismantas *et al.*^12^ who identified a slightly higher percentage of dasatinib-sensitive patients (30–40%) in their T-ALL cohorts. In addition to confirming their main findings, we expand on them in two different ways. First, we identified *LCK* tyrosine kinase as the potential prime target of dasatinib, whereas Frismantas *et al.*^12^ did not recognize any recurrent gene fusions, mutations or transcriptome profiles associated with dasatinib sensitivity; rather, they associated *SRC* expression and phosphorylation status with dasatinib sensitivity. They supported their hypothesis by reporting a positive correlation between dasatinib and SRC inhibitor KX2-391 responses in T-ALL patient samples.^12^ However, DsigDB identifies both SRC and LCK as targets of KX2-391. In our GEO-based gene expression data set and T-ALL cell lines, the expression of *SRC* was very low when compared with *LCK*, and there was no difference in the expression levels of T-ALL and normal T-cell samples (Supplementary Figure S1). A low level of *SRC* expression was also observed in the data set of Frismantas *et al.*^12^ Importantly, the *LCK*-mutant derivative of the Jurkat cell line failed to respond to dasatinib, thus strongly suggesting the central role played by LCK itself. We failed to detect an association between the phosphorylation status of LCK kinase and dasatinib sensitivity in T-ALL cell lines (data not shown).

Second, we show here that patients belonging to the TAL1 subgroup are the most likely to respond to dasatinib, although not exclusively. A high expression of *LCK* associates with the TAL1 subgroup, but TAL1 does not directly regulate *LCK* expression. As up to 60% of T-ALL patients belong to the TAL1 subgroup and only 30–40% respond to dasatinib, there is clearly a need for more accurate biomarkers to be identified.

Dasatinib is known to suppress proliferation of healthy T cells by LCK inhibition.^13, 14, 15^ We noticed a significantly higher expression of *LCK* in T-ALL samples compared with healthy T cells (a 1.46-fold change, *P*<0.001), and a higher sensitivity of T-ALL cells toward dasatinib compared with healthy bone marrow cells. Although we identified LCK as the main target of dasatinib, our results do not exclude the contribution by other kinases, including other Src family kinases. Many of the target candidates function in receptor signaling pathways related to cell proliferation and survival. LCK and FYN are components of the T-cell receptor (TCR) signaling cascade.^16^ During T-cell development, LCK is required for the normal development of thymocytes, whereas in mature T cells, FYN is capable of activating several TCR signaling pathways in the absence of LCK, including the Ras/extracellular signal-regulated kinase and phosphatidylinositol 3-kinase pathways.^16^ In knockdown experiments, we observed reciprocal feedback mechanisms between LCK and FYN (data not shown), in agreement with their known partially overlapping functions.

Dasatinib is a tyrosine kinase inhibitor that is currently approved for imatinib-resistant Philadelphia chromosome-positive (Ph+) chronic myeloid leukemia and the second-line treatment of Ph+ ALL. We report here the potential utility of dasatinib in the treatment of a subset of T-ALL. As our patient cohort is relatively small, further studies are needed to explore the biomarker findings and to deepen the mechanistic basis before embarking on animal and human studies.

## Figures and Tables

**Figure 1:**
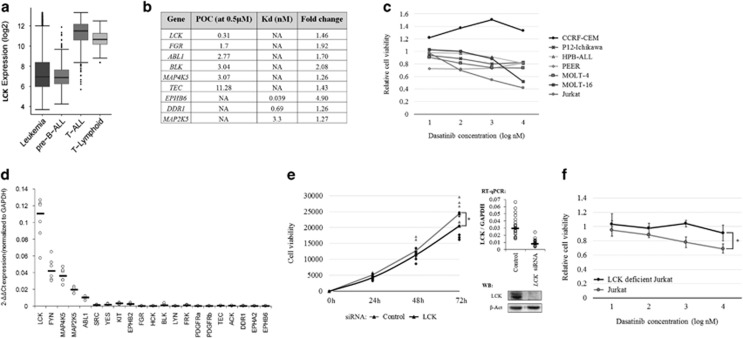
The *in silico* and *in vitro* results indicate dasatinib as a potential drug for T-ALL with *LCK* as its prime target. (**a**) *LCK* expression in different hematological sample groups: acute leukemia (*n*=4430), pre-B-ALL (*n*=1304), T-ALL (*n*=385) and T lymphoids (*n*=247). (**b**) The filtered list of targets of dasatinib from *in silico* screening. The list contains targets with a lower expression in normal cells (myeloid, B lymphoid and T lymphoid) in comparison with their leukemic counterparts; a significant expression difference between T-ALL and T-lymphoid samples (adjusted *P*<0.001 and a >1.25-fold change) and dasatinib is capable of inhibiting them with high efficiency. Percentage of control (POC) or Kd values were used to estimate the efficacy of dasatinib against its targets. The POC value indicates the percentage of remaining activity after inhibitor treatment in comparison with the untreated control sample. In addition to 0.5 μM, POC values at 100 nM concentrations were also available for LCK and ABL1, and they were 1 and 0, respectively. (**c**) Effect of dasatinib for cell viability in several T-ALL cell lines measured by alamarBlue assay after 72 h of incubation in 10-fold dasatinib dilution series (1–1000 nM). Values are relative cell viabilites in comparison with dimethyl sulfoxide (DMSO) control and results are median values from three independent experiments performed in triplicate, except for CCRF-CEM and HPB-ALL that are from two independent experiments. (**d**) The expression of 21 potential dasatinib targets in Jurkat cells. Bars indicate median values. (**e**) The effect of *LCK* knockdown for Jurkat cell proliferation measured in time series (0h, 24h, 48 and 72 h) with alamarBlue assay. Proliferation trend lines are drawn through median values. At time point 72 h, the proliferation had decreased by 14% in comparison with the mock-treated control (*P*=0.0289, Mann–Whitney *U*-test). The data consist of seven individual experiments performed in triplicate, and each time point is normalized to the 0 h time point. RT-qPCR and western blot results show the efficiency of *LCK* knockdown. (**f**) The effect of dasatinib on cell viability in the LCK-deficient Jurkat cell line in comparison with the normal Jurkat cell line measured by alamarBlue assay after 72 h of incubation in a 10-fold dasatinib dilution series (1–1000 nM). The difference between the two cell lines was statistically significant already at a 10 nM concentration (*P*=0.014, Mann–Whitney *U-*test). The values are relative cell viabilities in comparison with the DMSO control, and the results are the median values from three independent experiments performed in triplicate. The error bars indicate 95% confidence intervals.

**Figure 2:**
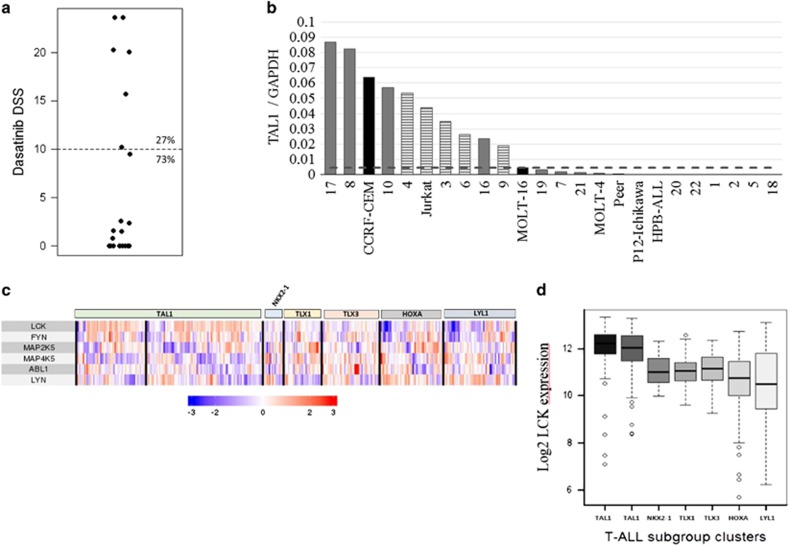
Dasatinib-sensitive subgroup of T-ALL samples. (**a**) Drug sensitivity scores (DSS) of dasatinib in a cohort of 22 patient samples. These DSS values are calculated from growth inhibition measurements after 72 h of treatment in a 10-fold dasatinib dilution series (0.1–1000 nM), and a DSS value of 10 was used as the threshold for dasatinib sensitivity. (**b**) The expression of the T-ALL subtype defining transcription factor *TAL1* in T-ALL patient samples and cell lines measured by RT-qPCR. The threshold for ectopic *TAL1* expression (dashed line) is defined by the expression of *TAL1* in SIL-TAL1 fusion-positive cell lines, indicated with black columns, and the striped columns represent the dasatinib-sensitive samples.^17^ Patient sample 4 was processed in a separate RT-qPCR batch. (**c**) The heat map of *LCK*, *FYN*, *MAP2K5*, *MAP4K5*, *ABL1* and *LYN* expression in a separate GEO-based T-ALL sample data set (*n*=385). Samples are clustered based on the expression of T-ALL subtype-defining transcription factors: *TAL1* (*n*=61+103), *NKX2-1* (*n*=18), *TLX1* (*n*=33), *TLX3* (*n*=51), *HOXA* (*n*=56) and *LYL* (*n*=63). (**d**) *LCK* expression in T-ALL subtype clusters. The difference between TAL1 clusters and any other cluster was statistically significant (*P*<0.001, Mann–Whitney *U-*test).
