# Supplementary material for: In silico and preclinical drug screening identifies dasatinib as a targeted therapy for T-ALL
Source: Blood Cancer J. 2017 Sep 8;7(9):e604–. doi: 10.1038/bcj.2017.87 (PMC5709756; doi:10.1038/bcj.2017.87)
Supplement: Supplementary Figures [file bcj201787x1.pdf]

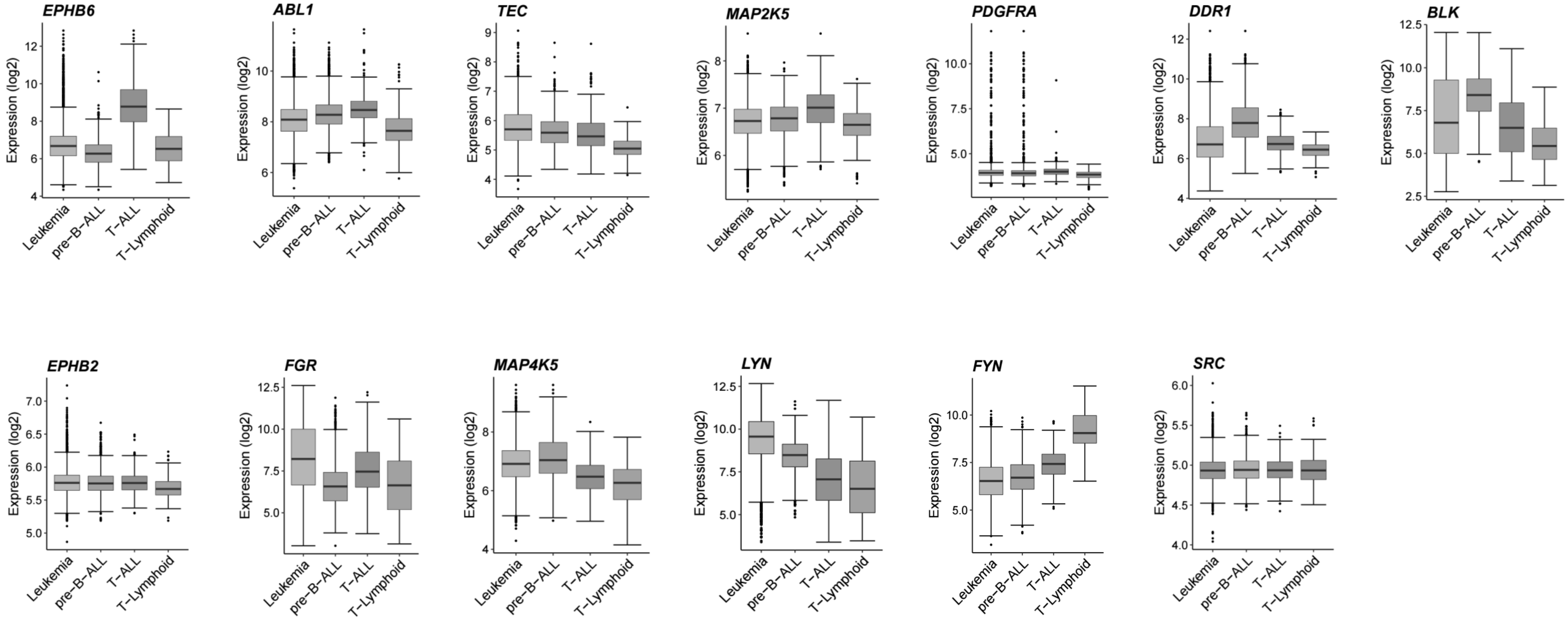

Supplementary Figure S1. The gene expression boxplots of several dasatinib targets in different hematological sample groups: acute leukemia (n=4,430), B-ALL (n=1,304), T-ALL (n=385), and T lymphoids (n=247).

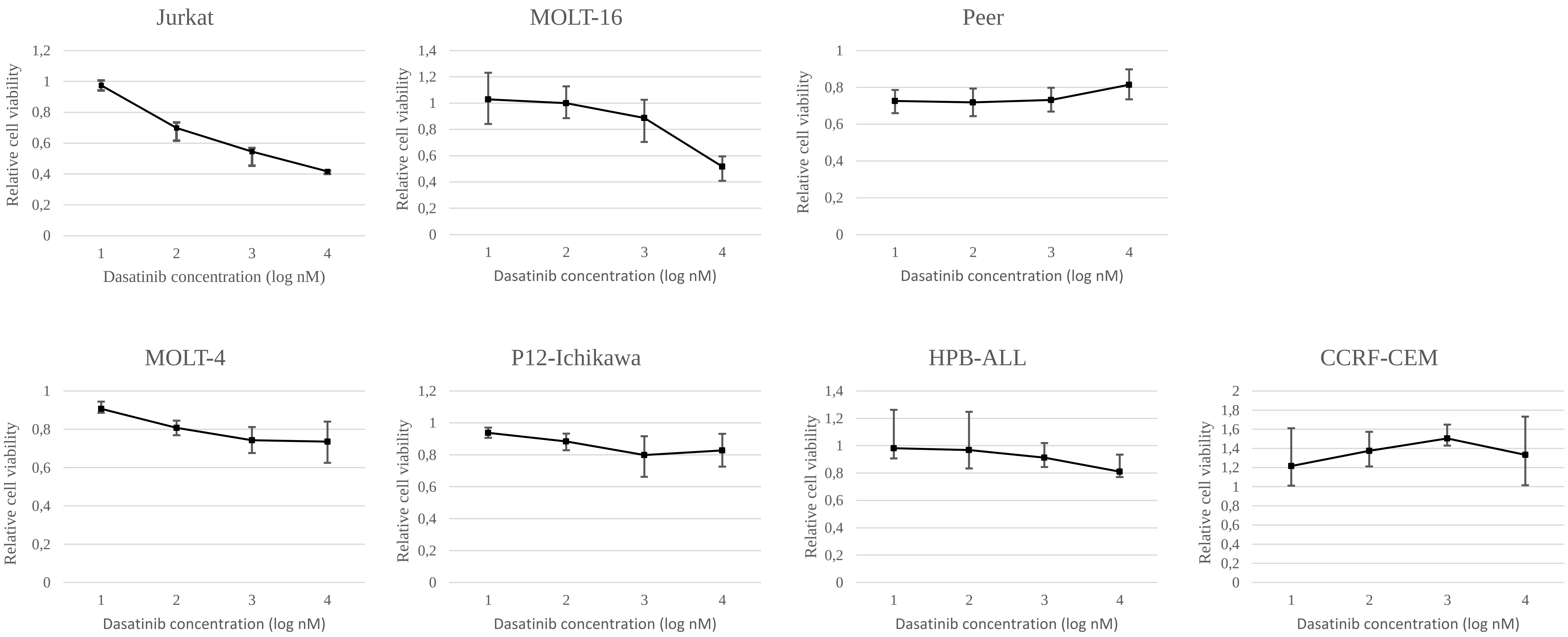

Supplementary Figure S2. The relative cell viabilities of dasatinib treated T-ALL cell lines measured by alamarBlue assay after 72h incubation in a 10-fold dasatinib dilution series (1–1000 nM). The values are relative cell viabilities in comparison to the DMSO control, and the results are the median values from three independent experiments (except for CCRF-CEM and HPB-ALL, which are from two independent experiments). The error bars indicate 95% confidence intervals.

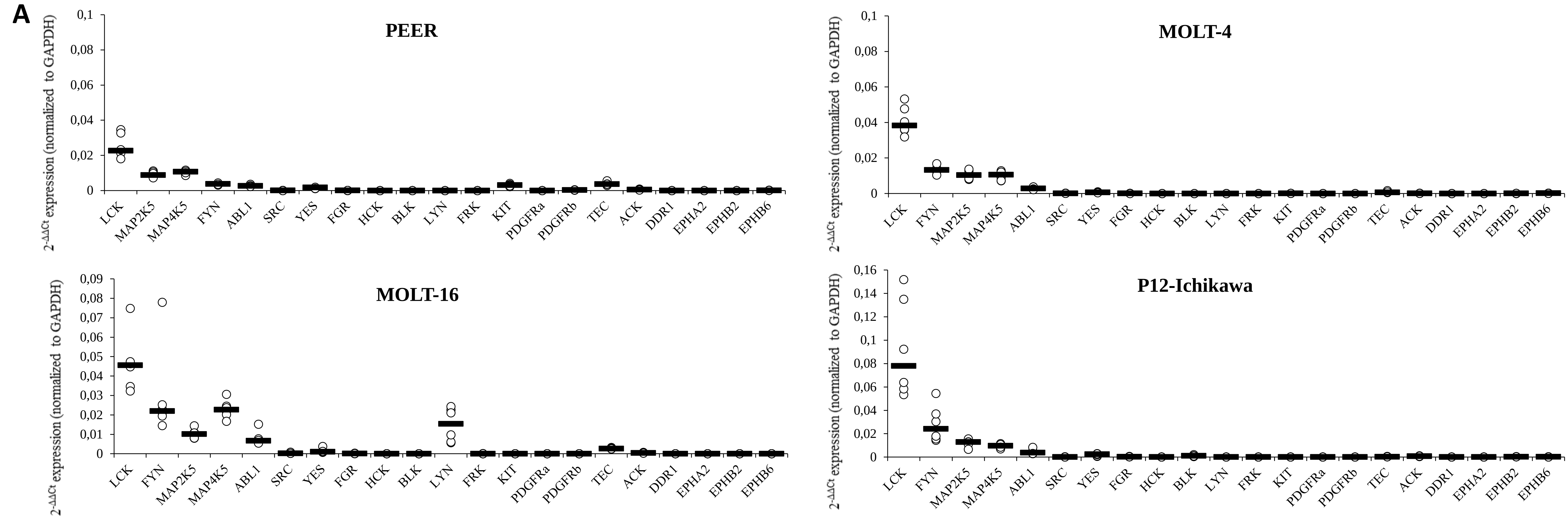

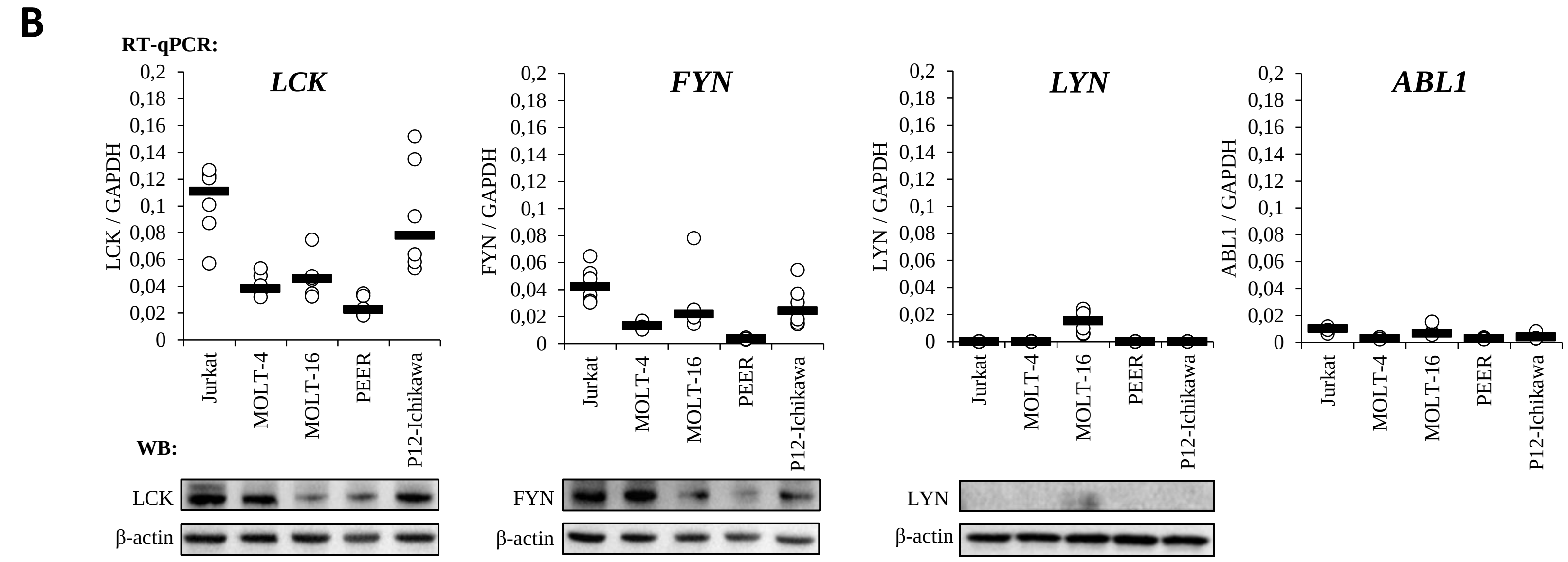

Supplementary Figure S3. A) The RT-qPCR of 21 known dasatinib targets in several T-ALL cell lines. The values are normalized to GAPDH housekeeping control, and the bars indicate the median values. B) The expression of interesting dasatinib-target candidates (*LCK*, *FYN*, *LYN*, and *ABL1*) in five T-ALL cell lines measured by RT-qPCR and Western blotting. Wt ABL1 expression was not detectable by the antibody that recognized the BCR-ABL1 fusion protein in the control sample (2862, Cell Signaling Technology), suggesting a low expression of the protein. The bars in the RT-qPCR plots indicate the median values.

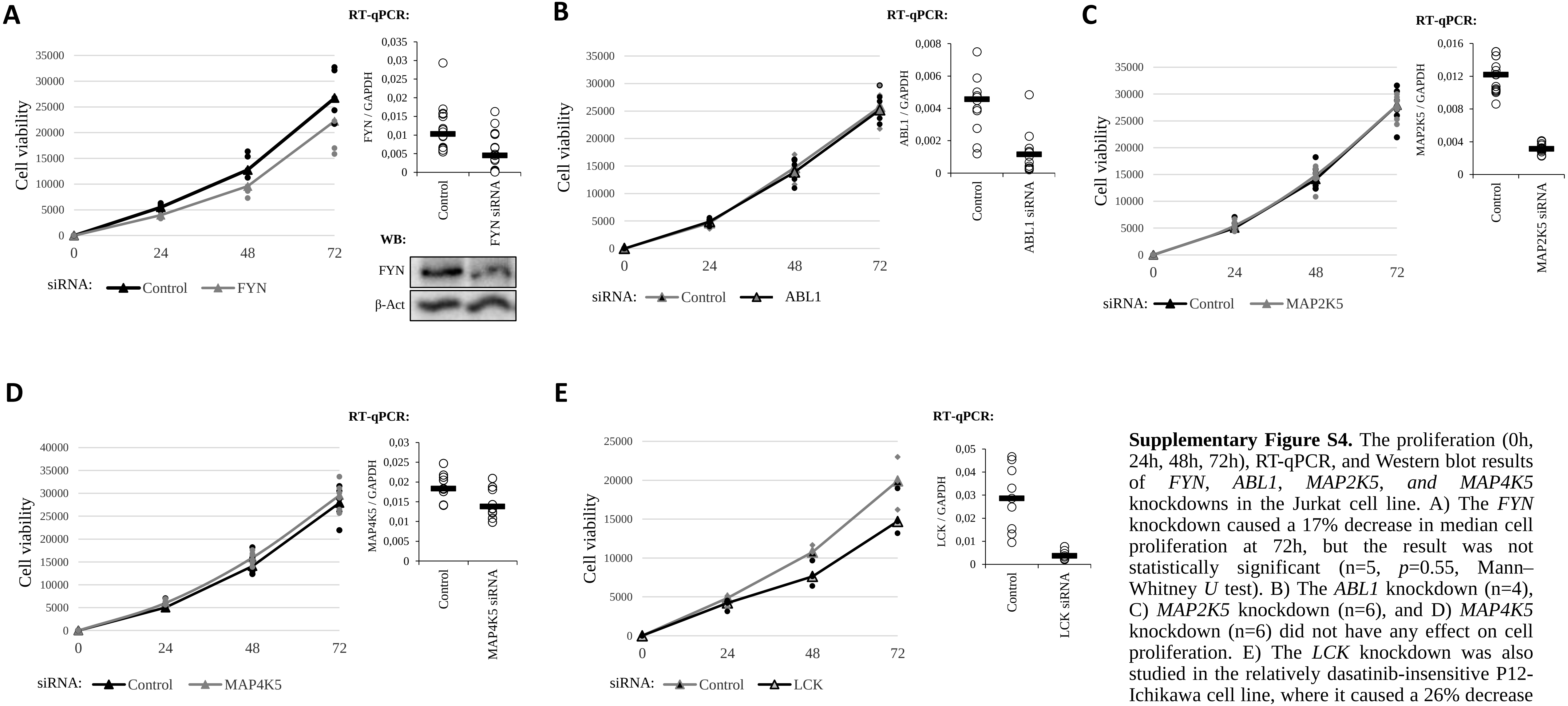

Supplementary Figure S4. The proliferation (0h, 24h, 48h, 72h), RT-qPCR, and Western blot results of *FYN*, *ABL1*, *MAP2K5*, *and MAP4K5* knockdowns in the Jurkat cell line. A) The *FYN* knockdown caused a 17% decrease in median cell proliferation at 72h, but the result was not statistically significant (n=5, *p*=0.55, Mann–Whitney *U* test). B) The *ABL1* knockdown (n=4), C) *MAP2K5* knockdown (n=6), and D) *MAP4K5* knockdown (n=6) did not have any effect on cell proliferation. E) The *LCK* knockdown was also studied in the relatively dasatinib-insensitive P12-Ichikawa cell line, where it caused a 26% decrease in median cell proliferation at 72h, but the result was not statistically significant (n=3, *p*=0.2, Mann–Whitney *U* test).

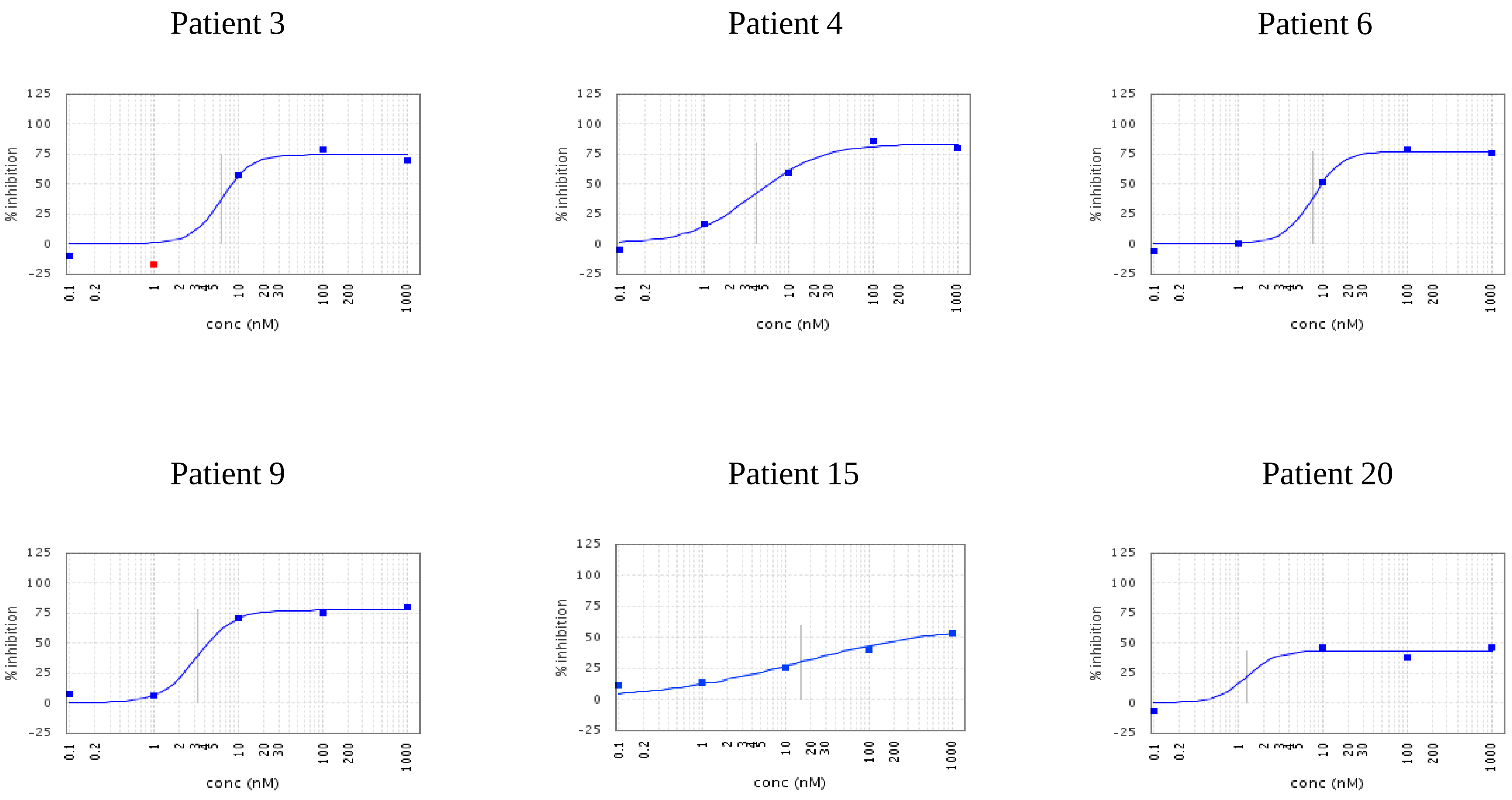

Supplementary Figure S5. Growth inhibition in dasatinib-sensitive patient samples after 72h of treatment. The data are normalized to negative (DMSO) and positive (benzethonium chloride) controls. The gray vertical lines indicate half maximal growth inhibition concentrations.

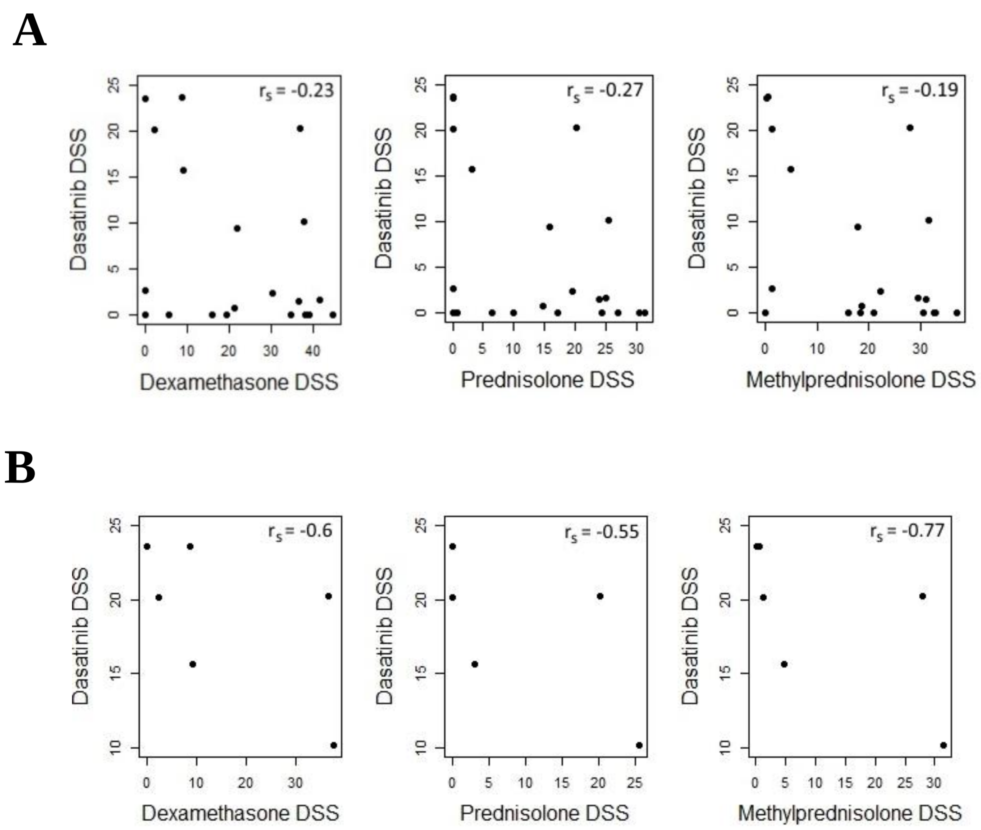

Supplementary Figure S6. A) The negative correlation in the effects of dasatinib and glucocorticoids (prednisolone and methylprednisolone) in a cohort of 22 T-ALL patient samples. B) The negative correlation in the effects of dasatinib and glucocorticoids if subsetting only dasatinib sensitive patient samples (n=6). Spearman’s correlation coefficients are shown.

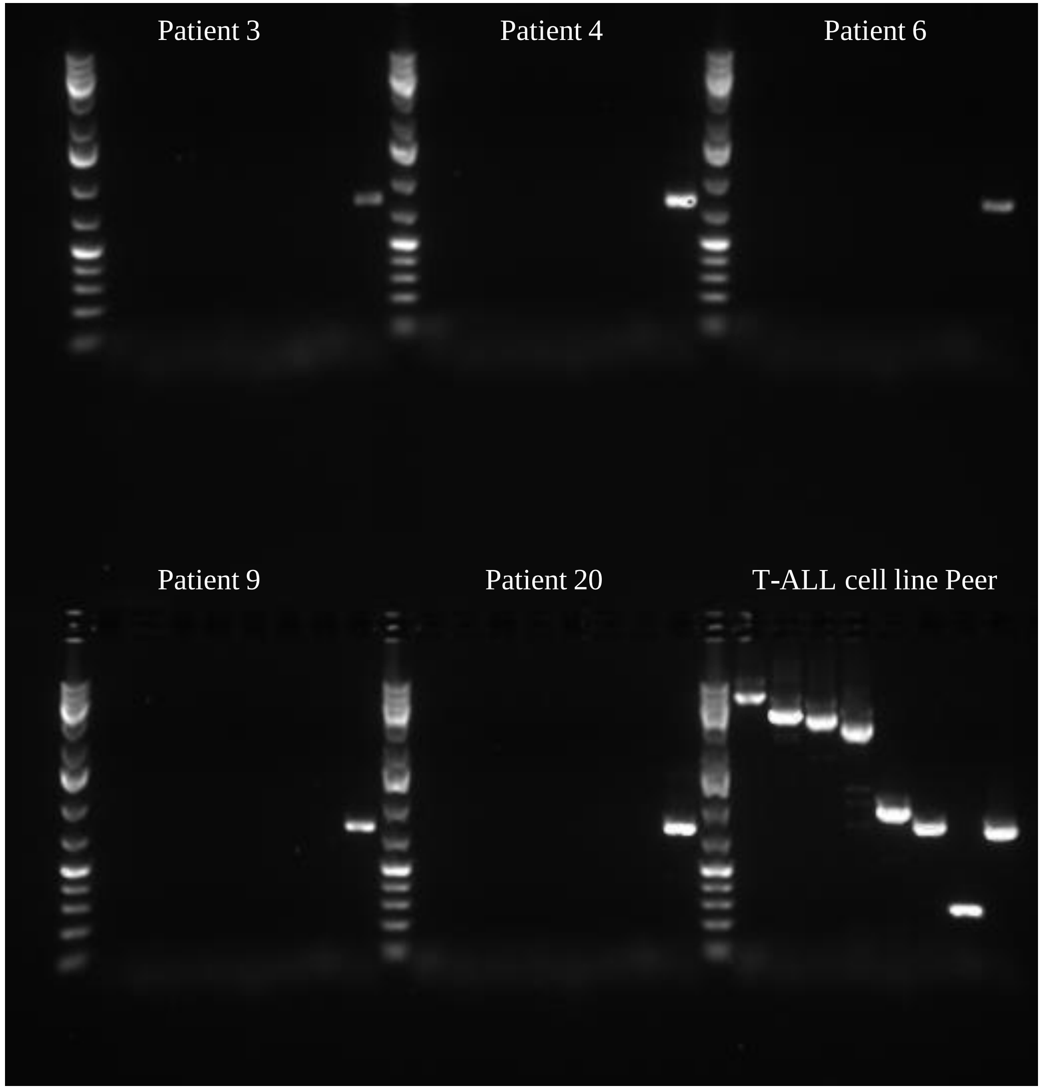

Supplementary Figure S7. The RT-PCR analysis of patient samples for *NUP214-ABL1* fusion. Lanes 1–7 present PCR primers for fusion with different breakpoints, and lane 8 presents the control reaction for RNA quality with PCR primers for *ABL1*. T-ALL cell line Peer was used as a positive control.

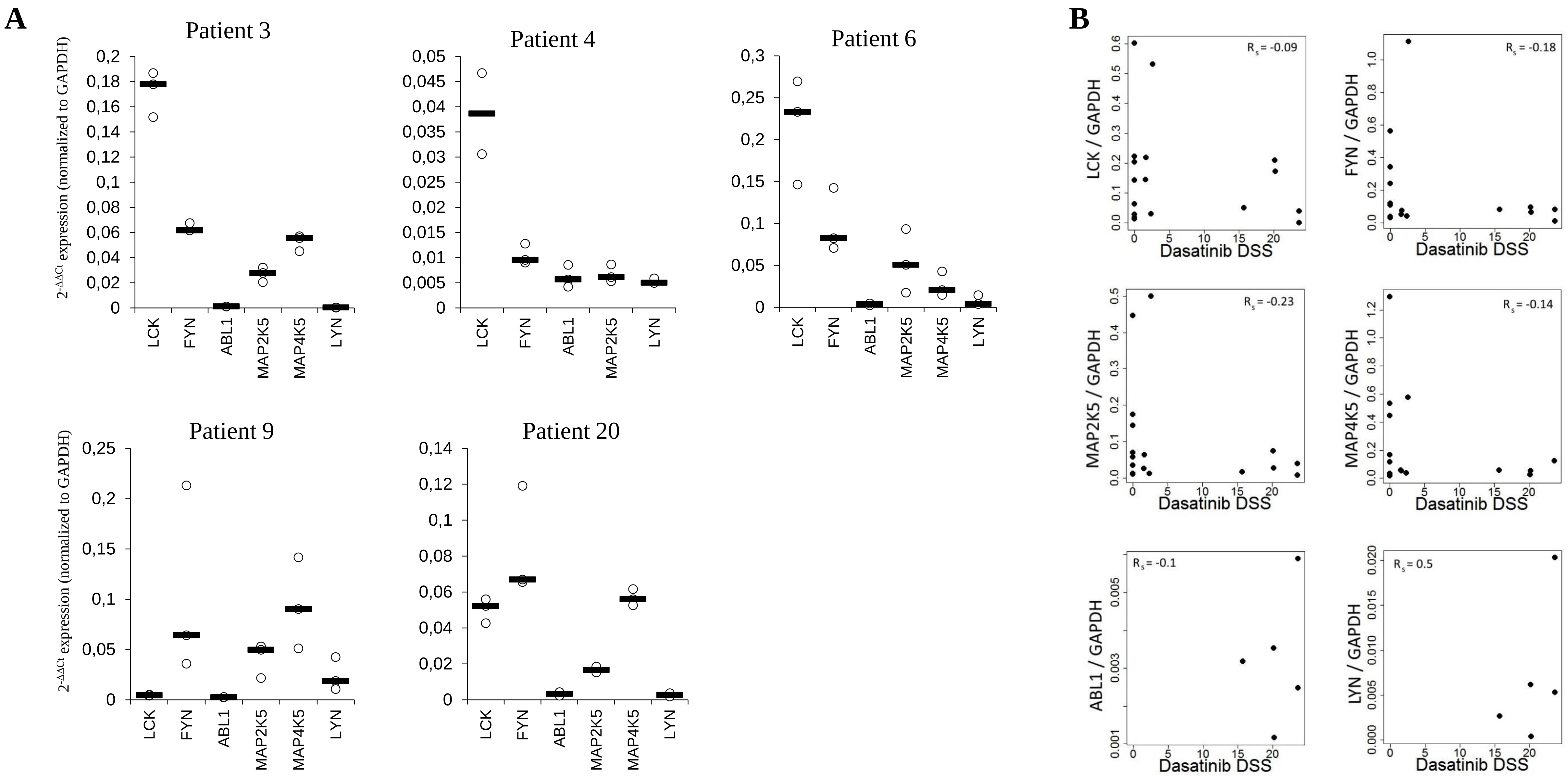

Supplementary Figure S8. A) The expression of *LCK*, *FYN*, *ABL1*, *MAP2K5*, *MAP4K5*, and *LYN* in five dasatinib-responsive patient samples. B) The correlation between Dasatinib DSS and *LCK*, *FYN*, *MAP2K5*, *MAP4K5*, *ABL1*, and *LYN* expression. *LCK*, *FYN*, *MAP2K5*, and *MAP4K5* expression is studied in both dasatinib-sensitive and insensitive samples (n=17). *ABL1* and *LYN* are studied only in dasatinib-sensitive patient samples (n=5). The *ABL1* and *LYN* of all samples and *FYN* of patient 4 were processed in a separate RT-qPCR batch.

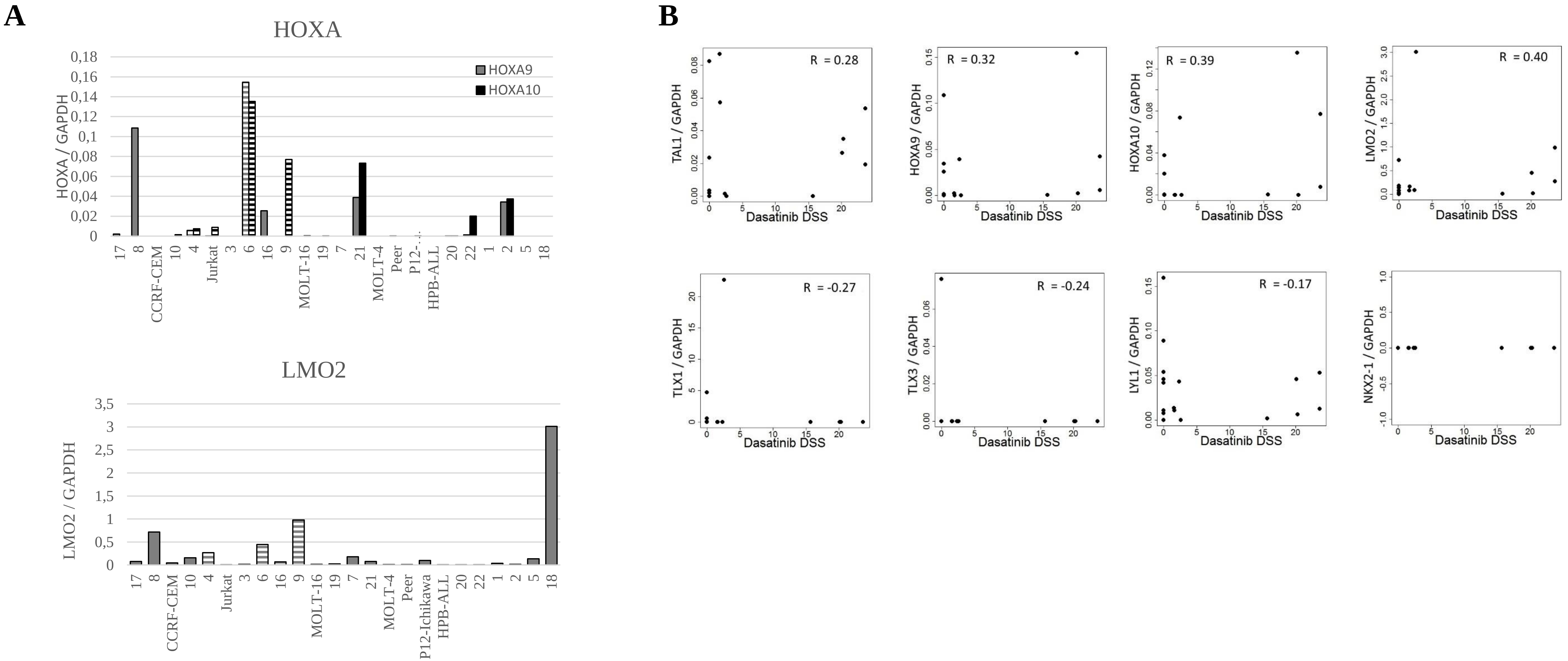

Supplementary Figure S9. A) The expression of T-ALL subtype-defining transcription factors *HOXA9/10* and *LMO2* in T-ALL patient samples, and the cell lines measured by RT-qPCR. The striped columns indicate the dasatinib-sensitive samples. B) The correlation between dasatinib response and expression of T-ALL subtype-defining transcription factors: *TAL1*, *HOXA9/HOXA10*, *LMO2*, *TLX1*, *TLX3*, *LYL1*, and *NKX2-1* in patient samples (n=17). Patient sample 4 was processed in a separate RT-qPCR batch, and none of the samples expressed *NKX2-1*.

A

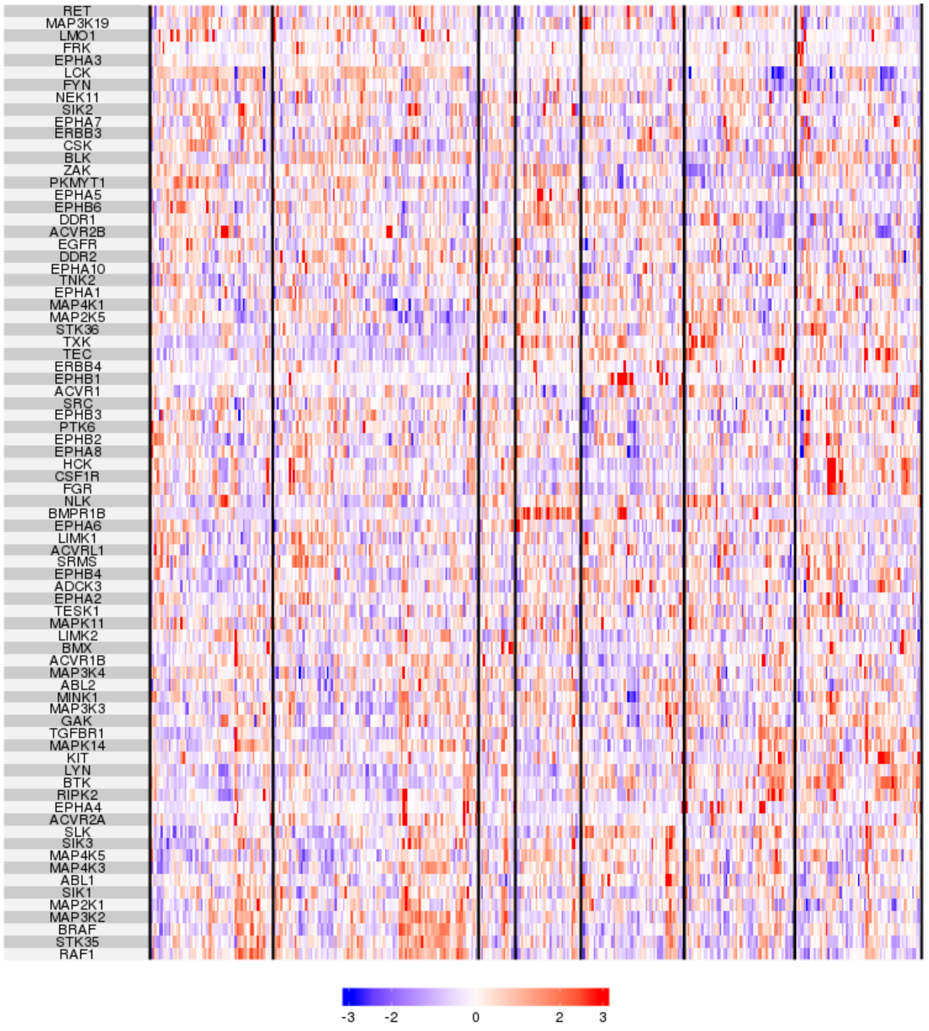

B

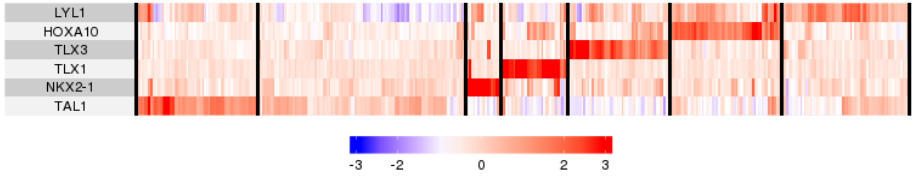

Supplementary Figure S10. A) Expression of 83 possible dasatinib targets in T-ALL subgroup clusters that are based on subtype-defining transcription factors: *TAL1* (n=61+103), *NKX2-1* (n=18), *TLX1* (n=33), *TLX3* (n=51), *HOXA* (n=56), and *LYL* (n=63). B) The heat map of T-ALL subtype clustering.

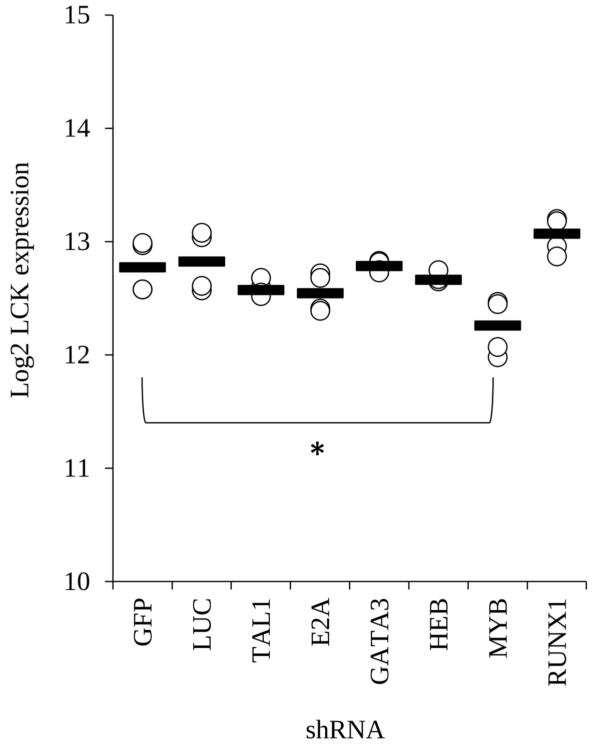

Supplementary Figure S11. *LCK* expression in the Jurkat cell line after shRNA knockdowns of *TAL1* and several other transcription factors related to T-ALL (data reproduced from Sanda et al. 2012, *Cancer Cell*). The only significant expression difference was observed between the *MYB* and *GFP/LUC* control knockdowns (*GFP* vs. *MYB*, p=0.021, Mann–Whitney U test).
