# Supplementary material for: In silico and preclinical drug screening identifies dasatinib as a targeted therapy for T-ALL
Source: Blood Cancer J. 2017 Sep 8;7(9):e604–. doi: 10.1038/bcj.2017.87 (PMC5709756; doi:10.1038/bcj.2017.87)
Supplement: Supplementary Tables [file bcj201787x2.docx]

**Supplementary Table S1.** Clinical information of patient samples. Highlighted samples were dasatinib responsive.

| Sample ID | Age at diagnosis | Sample (diagnosis/relapse) | Cytogenetics | Immunophenotype | Additional immunophenotype information |
| --- | --- | --- | --- | --- | --- |
| 1 | 28 | Diagnosis | NA | T-ALL | Mature |
| 2 | 40 | Relapse | NA | T-ALL | Cortical |
| **3** | **17** | **Diagnosis** | **46<XY,del(6)(q21q25)[4]/46,XY[16]** | **T-ALL** | **Cortical** |
| **4** | **50** | **Diagnosis** | **46,XY,t(11;14)(p13;q11)[4]/46,XY[16]** | **T-ALL** | **Cortical** |
| 5 | 72 | Diagnosis | 40~44,X,-X,del)3_(q21),-5,add(6),q2?3),add(7)(pl?),-8,- | T-ALL | Immature |
| **6** | **75** | **Diagnosis** | **46,XY** | **T-ALL** | **NA** |
| 7 | 16 | Diagnosis | NA | T-ALL | Immature |
| 8 | 13 | Diagnosis | NA | T-ALL | Cortical |
| **9** | **26** | **Diagnosis** | **46, XY, del(6q)** | **T-ALL** | **Cortical** |
| 10 | 63 | Diagnosis | 46,YY,t(1;14)(p32;q11), deletio 6q matching with T-ALL, add(11) | T-ALL | Mature |
| 11 | 18 | Relapse | Hyperdiploid 48, +8,+18 | T-ALL | NA |
| 12 | 55 | Diagnosis | hyperd.48; t(1,16), trisomies 10 and 19 | T-ALL | NA |
| 13 | 38 | Diagnosis | 46,XY,t(3;19)(p?21;p13),t(10;11)(p13;q14ü21)  .ish t(3;19)(E2A+;E2A),t(10;11)(WCP10+, WCP11+,MLL+; WCP11+,MLL-,WCP10+) | T-ALL | NA |
| 14 | 61 | Diagnosis | NA | T-ALL | NA |
| **15** | **27** | **Diagnosis** | **46,XY, del 6q21** | **T-ALL** | **NA** |
| 16 | 29 | Diagnosis | NA | T-ALL | NA |
| 17 | 23 | Diagnosis | 46,XY | T-ALL | NA |
| 18 | NA | NA | NA | T-ALL | NA |
| 19 | NA | NA | NA | T-ALL | NA |
| **20** | **NA** | **NA** | **NA** | **T-ALL** | **NA** |
| 21 | NA | NA | NA | T-ALL | NA |
| 22 | NA | NA | NA | T-ALL | NA |

**Supplementary Table S2.** SiRNAs for nucleofection

| Target gene | siRNA (OriGene) | Catalog No. |
| --- | --- | --- |
| ABL1 | ABL1 (ID 25) Trilencer-27 Human siRNA | SR300017 |
| FYN | FYN (ID 2534) Trilencer-27 Human siRNA | SR301682 |
| LCK | LCK (ID 3932) Trilencer-27 Human siRNA | SR302659 |
| LYN | LYN (ID 4067) Trilencer-27 Human siRNA | SR302752 |
| MAP2K5 | MAP2K5 (ID 5607) Trilencer-27 Human siRNA | SR303764 |
| MAP4K5 | MAP4K5 (ID 11183) Trilencer-27 Human siRNA | SR307671 |
| Negative Control | Trilencer-27 Universal Scrambled Negative Control siRNA Duplex | SR30004 |

**Supplementary Table S3.** Nucleofection conditions

| Cell line | Solution | Reaction program |
| --- | --- | --- |
| Jurkat | SE | CL-120 |
| P12-Ichikawa | SF | CA-137 |

**Supplementary Table S4.** RT-qPCR primer sequences

| Primer | Sequence 5’ 3’ |
| --- | --- |
| LCK forward | GCA TCC TGG AGC AGA GCG G |
| LCK reverse | GCT CTC GCT CTC CCG GAT G |
| ABL1 forward | GTG GCC AGT GGA GAT AAC ACT |
| ABL1 reverse | CAG ATA CTC AGC GGC ATT GCG |
| SRC forward | GAT CCG CAA GCT GGA CAG CG |
| SRC reverse | CAT CCA CAC CTC GCC AAA GC |
| YES forward | GCT GGT TTA ACA GGT GGT GT |
| YES reverse | CCA AAA TAC CAT TCT TCT GCC |
| FYN forward | GCT TCG GTG TGA CCT CCA T |
| FYN reverse | GAG CGG GCT TCC CAC CAA T |
| FGR forward | GGT GAC TGG TGG GAG GC |
| FGR reverse | CTT CAC ATG ATC GCC TCT GG |
| HCK forward | GGA CCA GAT GGT GGT CCT A |
| HCK reverse | TCG CAC GGA CAA AGA GTA GC |
| BLK forward | CCA GGT CAC TCG TCA CAG G |
| BLK reverse | CCC CTT CAT CCA GGC AGC |
| LYN forward | CCT GGA GGA GCA TGG AGA AT |
| LYN reverse | CAT CAC CAT GCA CAG GGT C |
| FRK forward | CCT GTG TGT CAA GCT GGG G |
| FRK reverse | GTG CCT CCC TCA GGA AGT C |
| KIT forward | GCA TTG TTC TGT GGA CCA GG |
| KIT reverse | GAA GTC ACC GTG ATG CCA G |
| PDGFRa forward | CTA TGT GCC AGA CCC AGA TG |
| PDGFRa reverse | CGG TGG CCT CAC AGA TAT AG |
| PDGFRb forward | CTT TGT GCC AGA TCC CAC C |
| PDGFRb reverse | GTC CCC AAT GGT GGT TTT GC |
| TEC forward | GGT CAT TTC AGT AGA GAC GTA C |
| TEC reverse | GGA CAC CAC TTC ACA GGA AAC |
| ACK forward | GGA TGA GTA AGG TGT TCA GTG |
| ACK reverse | CAC TTC ACA GCC ACA CTC AC |
| EGFR forward | GTG ACT GCT GCC ACA ACC A |
| EGFR reverse | GCC GTG ATC TGT CAC CAC A |
| DDR1 forward | CCA CAG CAG GTT GGA GAG CA |
| DDR1 reverse | CCT GAG ATC ACC TCC TGA CC |
| EPHA2 forward | CCA GGC AGG CTA CGA GAA G |
| EPHA2 reverse | GCA AGG CAT CGA CGC TGG |
| MAP2K5 forward | CCT GAA GGT GAA TAC TCG GG |
| MAP2K5 reverse | GCT TTG TAG ACT GTG CCT CC |
| MAP4K5 forward | GGA GCC TGG AGA TGA TTT TTC |
| MAP4K5 reverse | GCA AAT AGG CAA GAC CCT GTA |
| EPHB2 forward | CAC CGT CTG CCG AGG TTG T |
| EPHB2 reverse | GAT GGT TGT GCA GGG CAT G |
| EPHB6 forward | GAC AAG GCC TGC CAA GCC T |
| EPHB6 reverse | CCG ATG GAG GAC CAG TGC A |

**Supplementary Table S5.** RT-PCR primer sequences

| Primer | Sequence 5’ 3’ |
| --- | --- |
| NUP214 exon 2, forward | ACC CGG AGA TGA TCC CAA CAA AAT^1^ |
| NUP214 exon 20, forward | CAC CAA ATC CTT GCC CAA AGT ACC^1^ |
| NUP214 exon 23, forward | CAA TGC TTG CCA CGA AAA CC |
| NUP214 exon 28, forward | TCA CAC CAA CAC CGT CTT CT |
| NUP214 exon 29, forward | TGG GTT CAG CTT TTG CCA AGC T^1^ |
| NUP214 exon 31, forward | GGT TTC TTC AGT GGC CTT GG |
| NUP214 exon 34, forward | CTG GTT TTG GAT CAG GCA CAG GA^1^ |
| ABL1 exon 3, reverse | CCA TTT TTG GTT TGG GCT TCA CAC C^1^ |
| ABL1 exon 1, forward | GTA GCC AAA GAC CAT CAG CG |

**References**

1. Burmeister T, Gökbuget N, Reinhardt R, Rieder H, Hoelzer D, Schwartz S. NUP214-ABL1 in adult T-ALL: the GMALL study group experience. *Blood* 2006; 108:3556-3559
