# Supplementary material for: In silico and preclinical drug screening identifies dasatinib as a targeted therapy for T-ALL
Source: Blood Cancer J. 2017 Sep 8;7(9):e604–. doi: 10.1038/bcj.2017.87 (PMC5709756; doi:10.1038/bcj.2017.87)
Supplement: Supplementary Materials and Methods [file bcj201787x3.docx]

**Materials and methods**

1. ***In silico* drug screen**

A table of gene level details for Dasatinib was obtained from DSigDB (DSigDBv1.0 Detailed.txt). Data sources for drug in silico analysis were TTD, GSK, Kinome Scan, LINCS, MRC, RBC and Roche. Gene expression from total of 83 target genes was examined in 4430 leukemia samples and 929 normal samples. One tailed wilcoxon test, followed by Bonferroni adjustment of *P*-values and fold change was computed to compare normal myeloid samples for AML, B-lymphoid for pre-B-ALL and T-lymphoid for T-ALL. Therefore, genes highly expressed in cancer compared to normal cells are prioritized as drug target candidates. Similarly, the Wilcoxon test was used to test whether the drug target gene is expressed at higher level in cancer compared to normal erythroid, myeloid, B-lymphoid or T-lymphoid samples to assess if drug has off target effects in normal cells.

1. **Cell lines and cell culture**

Jurkat, J.Cam1.6, MOLT-16, P12-Ichikawa, HPB-ALL and CCRF-CEM cells were cultured in RPMI (Gibco, Thermo Fisher Scientific, Waltham, MA, USA), 2 mM L-glut, 100 U penicillin, 100 µg/ml streptomycin and 10% FBS (Gibco) at 37°C in 5% CO_2_. MOLT-4 and PEER cells were cultured in RPMI (Gibco), 2 mM L-glut, 100 U penicillin, 100 µg/ml streptomycin and 20% FBS (Gibco) at 37°C in 5% CO_2_. All cell lines, except J.Cam1.6, have been authenticated using services provided by Eurofins Genomics (Ebersberg, Germany) and all cells have been regularly tested and proven negative for the presence of mycoplasma. J.Cam1.6 cell line was purchased from American Type Culture Collection (Manassas, VA, USA) and all other cells have been purchased from Leibniz Institute DSMZ-German Collection of Microorganisms and Cell Cultures (Braunschweig, Germany)

1. **Patient material & consents**

Bone marrow (BM) and peripheral blood (PB) collection from all living patients was performed following a written informed consent, according to the protocols approved by local Institutional Review Boards and in accordance with the Declaration of Helsinki. Patient characteristics are summarized in Supplementary Table S1.

1. **Quantitative PCR**

Total RNAs from cell lines were extracted with PureLink™ RNA Mini Kit and On-Column PureLink® DNase Treatment Protocol was used when needed (Ambion® by Life Technologies, Thermo Fisher Scientific). Synthesis of cDNA was performed with iScript (BioRad, Hercules, CA, USA) and 100 - 500 ng of RNA was used as a starting material. SsoFast EvaGreen® Supermix (BioRad) was used for RT-qPCR reactions according to manufacturer's instructions. RT-qPCR runs were performed with BioRad CFX96^TM^ Real Time System (BioRad) with the following program: initial denaturation at 96°C for 30s, 39 cycles of denaturation at 96°C for 2 s, annealing at 60°C for 5 s, and plate read. RT-qPCR measurements were repeated from two to eight independent experiments performed in triplicate, except for patient samples which had one sample performed in triplicate, and the relative 2^-ΔΔC^_T_ method was used for quantification.^1^ All RT-qPCR primer sequences are listed in Supplementary Table S4.

1. **Western Blot**

Cells were lysed with M-PER reagent (Thermo Fisher Scientific) according to the manufacturer's instructions. Protein samples were subjected to 10–12% SDS–polyacrylamide gel electrophoresis and transferred to Amersham Protran Supported nitrocellulose membrane (GE Healthcare). Membranes were processed using standard procedures. Amersham ECL Reagent (GE Healthcare) was used for chemiluminescence reaction and chemiluminescence was detected with ChemiDoc^TM^ XRS+ using Image Lab^TM^ Software (Bio-Rad). PageRuler Plus prestained protein ladder (Thermo Fisher Scientific) was used as a reference for protein size. The following primary antibodies were used in this study: LCK (2714), FYN (4023), LYN (4576) and ABL1 (2862) (Cell Signaling Technology); β-actin (sc-1615, Santa Cruz Biotechnology, Santa Cruz, CA, USA). Horseradish peroxidase conjugated anti-rabbit (P0217) and anti-mouse (P0260) antibodies were used as secondary antibodies (Agilent Technologies, Santa Clara, CA, USA).

1. **Gene silencing and nucleofection**

Target genes were silenced using nucleofection and small interfering RNAs. Each target gene had gene specific kit constituting from three unique 27mer siRNA duplexes (OriGene, Rockville, MD, USA) (Supplementary Table S2). 20 µM stock solutions were resuspended from the lyophilized siRNAs in RNAse free siRNA Duplex Resuspension Buffer (SR30005). 4D-Nucleofector™ (Lonza, Basel, Switzerland) was used for siRNA transfections to target cells. For each nucleofection reaction 1 million cells were collected and centrifuged (90 x g, RT, 10 min). After centrifugation cells were resuspended directly to proper nucleofection solution with solution supplement and nucleofection reactions were carried out according to manufacturer’s instructions in single nucleocuvettes (Supplementary Table S3). Total siRNA concentration per reaction was either 300 or 600 nM depending on the follow up experiments. After nucleofection reaction prewarmed fresh media was added to the cuvettes and cells were transferred to 12-well plates. After 24 hours transfected cells were used in cell viability assays either. Knockdown levels were confirmed with RT-qPCR and western blot.

1. **Cell viability assays**

When working with transfected cells, 24 h after the nucleofector transfection fresh media was changed on the cells and 10 000 cells per well were plated in 96 wells. Cells were allowed to grow up to 72 hours. In all experiments, 10 µl alamarBlue reagent (Life technologies) was added to each well at each time points. Fluorescence was measured after 2 hours of incubation with excitation of 560 nm and emission of 590 nm using the Tecan fluorometer Infinite 200 (Tecan, Männedorf, Switzerland). Three technical replicates per sample were included in each proliferation experiment.

1. ***In vitro* drug treatments**

Dasatinib was used in 10-fold dilutions (1-1000 nM) and depending on the cell line, 10 000 - 50 000 cells were plated per well in 96 well plate in drug media or in DMSO control media. Cells were incubated at 37 °C, 5% CO_2_ and after 72h alamarBlue assay was performed as described in section 7. Measurements from drug treated samples were normalized to DMSO control sample to obtain relative cell viabilities. All experiments were performed with three or four technical replicates per sample.

Dasatinib (9052) was purchased from Cell Signaling Technology (Danvers, MA, USA) and was stored at –20 °C as a stock solutions in dimethyl sulfoxide (DMSO).

1. ***Ex vivo* drug treatments**

Mononuclear cells were isolated from bone marrow aspirates or peripheral blood samples by Ficoll density gradient (Ficoll-Paque PREMIUM; GE Healthcare, Little Chalfont, UK), washed, counted, and suspended in Mononuclear Cell Medium (MCM; PromoCell, Heidelberg, Germany) supplemented with 0.5 μg/mL gentamicin and 2.5 μg/mL amphotericin B or frozen in DMSO. One sample was cultured in RPMI media supplemented with 10% FBS, 12.5% condition media, penicillin/streptomycin and L-glut. Most of the samples were DMSO stored and therefore thawed and cultured in MCM overnight before the experiment.

For dose-response studies the media was changed and 10 000 cells/well were seeded to the prepared 384-well drug plates. After 72 h incubation at 37 °C, 5% CO_2_, cell viability was measured by adding 25 μl of CellTiter-Glo luminescent reagent (Promega) to each well and the luminescence signal measured with a PHERAstar FS plate reader (BMG Labtech). The data was normalized to negative control (DMSO only) and positive control wells (containing 100 μmol/L benzethonium chloride, effectively killing all cells). Dose-response curves were generated and DSS scores calculated as previously described.^2^ Dasatinib concentrations used in the experiments were 10-fold dilutions with range 0.1-1000 nM, whereas glucocorticoid (dexamethasone, prednisolone and methylprednisolone) concentrations were 1-10 000 nM.

1. **Detection of NUP214-ABL1 and SIL-TAL1 gene fusions**

RNA from patient samples was reverse transcribed to cDNA with Maxima Reverse Transcriptase and oligo(dT) primers (Thermo Fisher Scientific) using 100 ng of template RNA. Following cycler program was used: 30 min at 50 °C, 5 min at 85 °C. To detect NUP214-ABL1 fusion from cDNA templates, targeted PCR was performed using Phusion Hot Start II polymerase (Thermo Fisher Scientific) and primers listed in Supplementary Table S5. Seven pairs of primers were used to detect NUP214-ABL1 fusion, covering the known range of fusion breakpoints.^3^ Additionally, primers targeting ABL1 regardless of the sample’s fusion status, were used to control RNA quality. All primers were diluted to a final concentration of 10µM and following cycler program was used: 30s at 98 °C, 35 cycles of 10s at 98 °C, 20s at 66 °C and 210s at 72 °C, followed by final extension of 5 min at 72 °C. PCR products were run on 1% agarose gel with GeneRuler 1kb Plus DNA ladder as a reference (Thermo Fisher Scientific) and gel was imaged with ChemiDoc^TM^ XRS+ using Image Lab^TM^ Software (Bio-Rad).

In one patient sample, NUP214-ABL1 and SIL-TAL1 fusions were detected from RNA-seq data. Agilent Bioanalyzer RNAnano chip (Agilent) was used to evaluate the integrity of RNA and Qubit RNA –kit (Life Technologies) to quantitate RNA in samples. 1ug of total RNA was used for ScriptSeqv2™ Complete kit for human/mouse/rat (Illumina, Inc., San Diego, CA, USA) to ribodeplete rRNA and further for RNA-seq library preparation. SPRI beads (Agencourt AMPure XP, Beckman Coulter, Brea, CA, USA) were used for purification of RNAseq libraries. The library QC was evaluated on High Sensitivity chips by Agilent Bioanalyzer. Paired-end sequencing of RNAseq libraries was done using Illumina HiSeq technology (HiSeq 2000, Illumina, Inc., San Diego, CA, USA). In data analysis, FusionCatcher software with default parameters was used for fusion detection.^4^

1. **Microarray data clustering and analysis**

Normalized microarray data of T-ALL samples with transcription factor encoding genes TAL1, TLX1, TLX3, HOXA10, NKX2-1 and LYL1 was first transformed with Principal Component Analysis to reduce the effect of uninteresting variance in the data. Then, k-means clustering was performed with several choices of parameter *k* to find suitable clustering complementing the subgroups of T-ALL defined by chosen transcription factors. Seven clusters was found to be optimal in representing these subgroups.

1. **Statistical methods**

R software was used for all statistical analysis. P-values were calculated with Mann-Whitney U-test and correlation coefficients with Spearman method. Unless otherwise stated in the text, P-value < 0.05 was considered statistically significant.

1. **References**

1. Livak KJ, Schmittgen TD. Analysis of relative gene expression data using real-time quantitative PCR and the 2(-Delta Delta C(T)) Method. *Methods* 2001; 4: 402-8.

2. Pemovska T, Kontro , Yadav B, Edgren H, Eldfors S, Szwajda A, *et al*. Individualized systems medicine strategy to tailor treatments for patients with chemorefractory acute myeloid leukemia. *Cancer Discov* 2013; 3: 1416-1429

3. Hagemeijer A, Graux C. ABL1 rearrangements in T-cell acute lymphoblastic leukemia. *Genes Chromosomes Cancer* 2010; 49: 299-308.

4. Nicorici D, Satalan M, Edgren H, Kangaspeska S, Murumägi A, Kallioniemi O, *et al*. FusionCatcher – a tool for finding somatic fusion genes in paired-end RNA-sequencing data. *bioRxiv* 2014: 011650
